# Encapsulation of Nutraceuticals in Yoghurt and Beverage Products Using the Ultrasound and High-Pressure Processing Technologies

**DOI:** 10.3390/foods11192999

**Published:** 2022-09-27

**Authors:** Mayumi Silva, Mayur Raghunath Kadam, Dilusha Munasinghe, Akalya Shanmugam, Jayani Chandrapala

**Affiliations:** 1 School of Science, RMIT University, Bundoora, VIC 3083, Australia; 2 Department of Biosystems Technology, Faculty of Technology, University of Sri Jayewardenepura, Pitipana 10206, Sri Lanka; 3 Food Processing Business Incubation Centre, National Institute of Food Technology, Entrepreneurship and Management, Thanjavur 613005, India; 4 Department of Food Science and Technology, Faculty of Applied Sciences, University of Sri Jayewardenepura, Nugegoda 10250, Sri Lanka; 5 Centre for Excellence in Non-Thermal Processing, National Institute of Food Technology, Entrepreneurship and Management, Thanjavur 613005, India

**Keywords:** yoghurt, beverages, ultrasound (US), high pressure processing (HPP), encapsulation

## Abstract

Dairy and beverage products are considered highly nutritious. The increase demand for added nutritional benefits within the food systems consumed by the consumers paves the pathway towards fortifying nutraceuticals into these products. However, nutraceuticals are highly unstable towards harsh processing conditions. In addition, the safety of dairy and beverage products plays a very important role. Therefore, various heat treatments are in practice. As the heat-treated dairy and beverage products tends to illustrate several alterations in their organoleptic characteristics and nutritional properties, the demand for alternative non-thermal processing technologies has increased extensively within the food industry. Ultrasound and high-pressure processing technologies are desirable for this purpose as well as a safe and non-destructive technology towards encapsulation of nutraceuticals into food systems. There are benefits in implementing these two technologies in the production of dairy and beverage products with encapsulants, such as manufacturing high-quality products with improved nutritional value while simultaneously enhancing the sensory characteristics such as flavour, taste, texture, and colour and attaining the microbial quality. The primary objective of this review is to provide detailed information on the encapsulation of nutraceuticals and mechanisms involved with using US and HPP technologies on producing encapsulated yoghurt and beverage products.

## 1. Introduction

Consumers are looking for customized food products that will help to meet their unique nutritional and personal health goals. As they are looking for more healthy food options, incorporation of functional ingredients or bioactives that have many health benefits to dairy and beverage products is a good strategy. Dairy and beverage products are frequently consumed and easily approachable by the consumers around the world. Among the secondary dairy products, yoghurt is considered as one of the most highly nutritious and functional food products. Beverages on the other hand has attained more recent attention due to busy lifestyle of consumers who sought convenience and extra nutritional benefits. 

The history of yoghurt has been started around 8000 years BC in Egypt. Yoghurt was resulted from an accidental fermentation due to lactic acid, which led to acidification of milk followed by coagulation. Though yoghurt is one of the oldest and traditional fermented milk products, it undergoes continuous changes to fulfil the demand in the market. There are two basic yoghurt products, which are set type and stirred type yoghurts. *Streptococcus thermophiles* and *Lactobacillus bulgaricus* are the two main lactic acid bacteria (LAB) responsible for the production of yoghurt. The organoleptic properties of yoghurt rely on three main factors, (i) the composition of the raw milk, (ii) food additives and (iii) production technique [1]. There were certain modifications made to the yoghurt products to improve the texture, nutritional properties and aroma such as the production of plain, sweetened and flavoured yoghurts in the form of drinks, frozen and concentrated. The nutritional properties were modified by the changes in fat and sugar concentrations [2]. Apart from these developments, the addition of healthy elements such as probiotic strains, vitamins, phytosterols have been introduced into yoghurt. This is done through encapsulation as these healthy elements are very unstable for processing. 

Similarly, ancient civilizations had their own preferred fruit juice combinations, although the commercial exploration of fruit-based beverages happened much later. Nowadays there are variety of products commercially available such as nectar, fruit juice, smoothie and drink with or without the addition of nutraceuticals. In addition, non-thermal processing technologies are implemented to produce encapsulated nutritionally sound beverages. However, all these beverage products are perishable in nature that have high chances of spoilage rate due to microorganisms, thus the safety of these products is vital. In order to maintain the quality of the products, various heat treatments are employed in practice. 

The thermal technology is a quite high efficiency treatment where the pathogenic microorganisms which are the main cause for the spoilage are inactivated. But due to the application of direct heat on the products, it causes irreversible modification of the colour, aroma and flavour along with loss of nutritional composition [3]. Therefore, to minimize these losses, Ultrasonication (US) or High pressure processing (HPP) are introduced which are eco-friendly and helps in inactivating the microorganisms without affecting the original quality of the food products. These technologies have advantages like producing high quality products with better nutritive value and enhanced sensory attributes like flavour, taste, texture and colour. It also includes some disadvantages like it is technically tough to apply into practice, quite costly, involves specialized equipment setup and trained personnel. The outcome of the product depends on the processing conditions, systems used, nutraceuticals used and the composition of the food products. 

The Ultrasound technology consists of the sound waves originating from the oscillated molecular movements in the medium of propagation. The ultrasonic waves will have the very high frequency of about 20 kHz which is not bearable by the human ear. These waves are classified into two different categories based on their frequency range—Low Intensity Ultrasonic waves (LIU) and High Intensity Ultrasonic waves (HIU) [4]. The High-Pressure Processing technique of foods is exhibited by applying the pressure of about 100–1000 MPa to the products by maintaining the temperature from 0–100 °C throughout the process. The pressure and time of exposure depends upon the type and state of the food. US and HPP technologies have proven to create encapsulating particles and uniform food products with specific functional and physical characteristics. 

The purpose of any food processing technology is to produce good quality products and to extend their shelf life by acting as a preservative technology while maintaining all the better sensorial attributes. The main objective of this review is to review the major applications and mechanisms of using US technologies towards producing yoghurt and beverage products encapsulated with nutraceuticals. Furthermore, advantages and disadvantages encountered will be discussed in detail.

## 2. Ultra-Sonication

In recent years, the food and dairy industry has been considerably using ultra-sonication, due to the increased awareness of its benefits and applications. Ultrasounds are the sound waves which has high intensity and frequency above the 18 kHz. There are two types of ultra- sonication, the high frequency—low power which operates at ~1 W/cm^2^ and 0.1–20 MHz and low frequency—high power operating at 10–1000 W/cm^2^ 18–1000 kHz. The imaging and diagnostic form that leaves the most food systems unchanged uses low power form while, the other operations in the food industry which causes physical disruption uses high power form [5]. The main principle of ultrasound is acoustic cavitation where small bubbles grow and collapse in the liquid. Transient cavitation and stable cavitation are the two main phenomena. In transient cavitation, growth of bubbles occurs in less acoustic cycle to fall into their resonance size and breaks into smaller bubbles due to their aggressive character of the collapse. In stable cavitation, once the bubble fall into resonance size, bubbles are rapidly collapsed and grown due to their less aggressive character. They can be seen ultrasound more than 200 kHz. The combination of these effects aids in better use of the ultrasound technologies for processing and other applications in dairy industries [6]. The prominence of this technology is to focus on improving processing efficiency and product quality by physical effects, destruction of microorganisms, and increasing the shelf life of the product as the other non-thermal technologies do [6]. The bubbles formed during acoustic cavitation on collapse produce tremendous turbulence and shock waves, which generate high temperatures and even leading to chemical changes [7]. The ultrasound can be used alone or in in combination to increase its effectivity, such as thermosonication (heat and ultrasound), manosonication (pressure and ultrasound), and manothermosonication (heat, pressure, and ultrasound), which is helpful to improve the product life [8]. The mechanism involved in inactivating the microorganisms and pathogens. When the cavitation bubble collapses asymmetrically, it leads to the rush of the liquid jet through the collapsed bubble centre. The microorganisms with the hydrophobic surfaces promote the cavitation of the bubbles on to the surface leading to severe damage of the cell membrane or cell wall and the erosion of cell wall takes place with the effects of the micro streaming, which results in the inactivation of the pathogens and microorganisms [9]. The localized heat produced during the process leads to production of free radicals causing the damage to the DNA which in turn leads to cell membrane thinning. All the above-mentioned effects due to the US treatment leads to the successful inactivation of microorganisms in the processed milk products. Also, the thickness and smoothness of the bacterial capsules plays a crucial role in deactivating the microorganisms by acoustic cavitation when high power of US are used [10].

The ultra-sonication system has 3 parts: frequency generator, transducer, horn or sonotrode. The sound waves in the material are generated that are caused due the contact with the vibrational energy that is transmitted by the sonotrode and is transferred to it by the transducer from the frequency generator. The temperature of the treated material is increased when the ultrasound energy is converted into heat. The quantity of heat produced, which depends on the output power of the sonicator and the length of the treatment, is used to quantify the actual power input of the sample. The area of the sonotrode and the equipment’s power output define the power density (W/cm^2^), whereas the power source influences power and wave amplitude. The effectiveness of ultrasonic treatment is determined by the total energy input for the volume of the product (kW h/L) and the power or energy intensity (W/cm^2^). The volumetric ultrasonic energy density (W/cm^3^) is used when the volume of the treated liquid surrounding the sonotrode is taken into account. However, because they are scale-independent, these two factors are solely employed for scaling up [11].

The application of ultrasound in the dairy has various advantages such as, the ultrasound waves are non-toxic and safe to apply on processing of food products. The loss of taste in the food can also be reduced and maintain the quality of the product. By combining the ultra-sonication with another non-thermal technologies, the inactivation of microbes can be carried out more effectively. It is cost efficient, easy to operate and has efficient power output. The use of ultrasound has also showed some limitations such as, it requires extra energy input, and the ultrasound generates physicochemical effects that leads to quality damage of food products through the production of off-flavours, alterations in physical properties and deterioration of components.

## 3. High Pressure Processing (HPP)

HPP technology has been widely used worldwide at industrial levels which helps in preserving wide range of food products in the absence of heat treatment and/or chemical preservatives. The dairy and beverage products are sensitive to changes organioleptic properties with thermal treatments, but HPP does not allow the products to obtain such changes as HPP eliminates the use of high temperature conditions [12]. Even though HPP is an efficient and globally recognised method of preservation for fruits, vegetables, fish, meat products, and other ready-to-eat meals, very limited applications are in use within the food industry. Modes of batch and semi-continuous processing, with restricted processing modes capacity for production, failure to effectively remove bacterial spores, and the high cost of equipment are big obstacles prevail of using HPP within food processing. Employing food safety regulations are needed, however is lacking due to its varying effectiveness in microbial reduction based on the type of the food product and its food composition. This needs to be addressed and must set certain limits for microbiological and quality scale to ensure the food is safe and quality assured for the consumers. However, it can be employed in combination with other technologies to speed up the processing steps and lowers the labour cost. 

HPP is based on two principles. One is Le Chatelier’s principle where it states that the system will try to counteract when any changes in a condition are applied to the system and the equilibrium is stored. Secondly, Isostatic principle (Pascal’s Law) which states that the pressure in the system is transmitted instantaneously and uniformly throughout the given sample in the system meaning the HPP is independent of the volume. Therefore, the size and dimensions of the products are irrelevant. The typical pressure range in a HPP setting is 300–600 MPa for 1–30 min. The temperature of the chamber for HPP is normally held below 40 °C, although some applications involve high temperature conditions. HP interferes with noncovalent bonds like ionic and hydrophobic bonds but has no effect on the covalent bonds. Large biomolecules, such as proteins and polysaccharides, are thus affected by changes to their secondary, tertiary and quaternary structures, but functional groups are not commonly affected. Since the compounds of colour, flavour and vitamins are small molecules HPP has no effect on these elements of the food [8].

The pressure applied spreads across the food instantaneously and evenly (based on the theory of Pascal’s), which is independent of the food mass’s size and geometry. After the pressure is removed, the compressed product then returns to its original form. Pressurization is followed by a uniform rise in temperature known as adiabatic compression of heat. The adiabatic heating level can vary significantly, and it increases to over 5 °C/100 MPa at 90 °C for water at pressures about 200 MPa’s [13]. Upon pressure release, the adiabatic heating is completely reversed. While this rise in temperature is relatively small, it may significantly contribute to the overall effectiveness of the microorganisms phase and has considerable implications. Food is therefore treated equally throughout and no particle escapes from the treatment. Furthermore, pressure gradients that would lower the efficiency of processing are not produced. This stands in comparison to technologies for heating, which frequently contribute to temperature gradients and uneven heating. Thus, HPP lowers the risk of under processing or inducing overheating of certain portions of the food.

## 4. Yoghurt and Yoghurt Products

Yoghurts are mainly made from milk, and starter cultures where stabilizers, emulsifiers, flavours, sweeteners are sometimes added during the commercial production. The milk type used depends upon the type of the yoghurt. For instance, the regular yoghurt is made with the whole cream milk, while low fat or non-fat yoghurts are made with skimmed milk. The fat content in the yoghurt is adjusted by adding cream and the total solids are improved by adding protein concentrates. The texture and body of the yoghurt are improved by the addition of stabilizers into the treated milk. After standardizing the fat and protein contents, the milk is subjected to heat treatment at 90 °C to 95 °C for 3 to 7 min, homogenization at 20 to 25 MPa at 70 °C, deaeration at 70 °C and cooling the milk to reach the incubation temperature of ~42 °C [14,15].

### 4.1. US Applications in Yogurt Products

The application of high intensity ultrasound on yogurt works on three key areas: effects on homogenisation, effects on the fermentation process and effects on the rheological and sensorial characteristics. The size of the milk fat globule is generally ranged within 1–10 μm and is surrounded by the milk fat globule membrane [16]. Native milk fat globules tend to aggregate with each other due to the interfacial tension and the agglutinins reactions [17]. It adversely affect the appearance and textural properties of yoghurt due to the possible formation of a milk fat layer during incubation and storage [18]. Thus, homogenisation reduces the size of the fat globules present in milk to <2 µm and consequently preventing the coalescence of fat globules during the fermentation process. This ensures the uniform distribution of fat globules within the protein matrix during the gelation process. Moreover, whey proteins and caseins effectively attach to the fat globule membrane during the homogenisation process [19,20]. 

Ultrasonic homogenisation known to be an effective way of homogenization as it is highly significant in reducing the size of fat globules [21,22,23] compared to the conventional homogenisation. Conventional homogenisation process is carried out with high pressure techniques utilising the turbulence and cavitation forces to disintegrate the fat globules [24] and then get stabilized by casein micelles, casein fragments and some whey proteins [25]. During the ultrasound-assisted homogenisation, physical forces presented by acoustic cavitation, especially turbulence and shear totally disintegrates the milk fat globule membrane and alter its composition and structure [23,26,27]. The newly formed fat globule membrane is wrapped with casein particles with their non-polar part [28] and with casein micelles [29]. In addition, some of the original milk fat globule membrane remains on the globule surface in minor quantities. These modified fat globule structures prevent the coalescence of fat globules [30] which thereby provides more stable base for the yoghurt production. Even though this is the scientific mechanism behind the ultrasound-assisted homogenisation process, various factors such as ultrasound conditions (frequency, power and time), experimental conditions (temperature and pressure) and characteristics of the milk base affect the homogenization efficiency of the yoghurt base.

Wu et al. (2000) [18] investigated the effect of ultrasound power and exposure time on the homogenisation efficiency. The pasteurized milk was treated under 20 kHz ultrasound at power levels of 90 W, 225 W, and 450 W for 1, 6, and 10 min and measured the homogenisation efficiency in terms of the size of the fat globules. The highest power level of 450 W provided the better homogenisation efficiency even at 1 min of sonication and increasing the sonication time up to 6 min resulted very small fat globules (<2 μm) and a uniform dispersion. In order to obtain the higher efficiency from low power levels, high exposure times were required due to the lesser cavitational effects provided by low power compared to high-power levels. Similarly, increase in efficiency of homogenisation process was observed with increase in applied sonication power where the highest applied power of 315 W provided higher homogenization efficiency at the longest treatment time of 6 min [31]. Moreover, size of the fat globule can reduce to as smaller as <1 μm using ultrasound at >300W power levels for 10 min [27]. For instance, increasing the power level up to 750 W resulted the fat globule size of 0.58 μm. Thermosonication of the yoghurt base with the combination of ultrasound (24 kHz, 400 W for 10 min) and increased temperature (72 °C) enabled the size reduction of fat globule up to 0.4–0.6 μm which was 2.5 fold reduction in comparison to the fat globule size obtained from conventional homogenisation of milk [28]. Therefore, it can be summarised that increasing the power and time of the sonication treatment under controlled conditions provided a more efficient homogenisation process by reducing the size of the fat globules. As shown in most of the studies, the power levels > 300 W were highly effective in size reduction of fat globules up to <1 μm. 

Some of the common issues associated with the fermentation of milk products is the longer fermentation times, insufficient fermentation, requirement of high quantities of culture and growth of unwanted microorganisms. These drawbacks adversely affect the quality and sensory properties of the final product and might increase the cost of production. Research findings suggest that the application of ultrasound to milk during the yoghurt production may facilitate the fermentation process by shortening the fermentation period, ramping up the live cell count, eliminating undesirable microorganisms, activating starter organisms more effectively, and enhancing the textural qualities. Efficiency of the ultrasound-assisted fermentation is depended on at which stage the ultrasound is applied to milk such as before, during or after the fermentation. Similarly, sonication parameters also play an important role where most of the beneficial effects were found to be prominent in higher ultrasound amplitudes of 90–225 W [18] or 150–750 W [32] under 20 kHz of frequency. 

**Figure 1:**
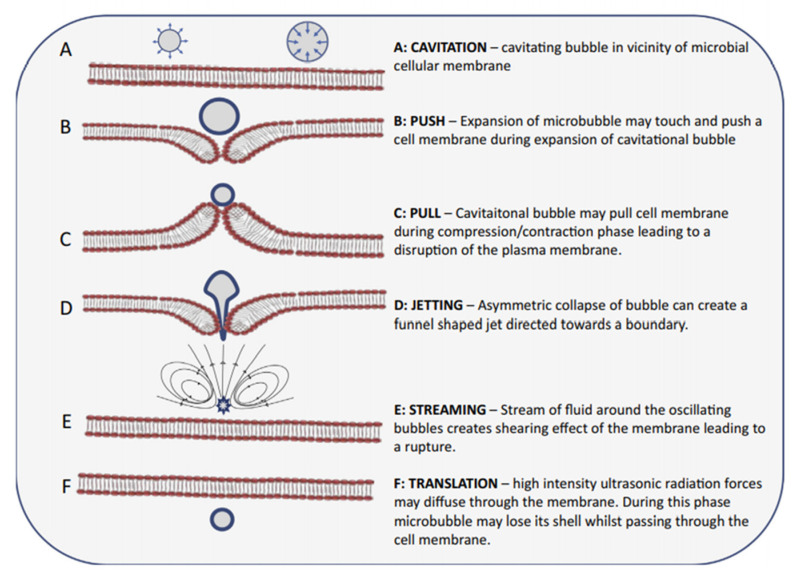
Sonoporation mechanism occurred during the ultrasound-assisted microbial inactivation process. Reproduced from [36] with permission from Elsevier, 2022.

It is well known that ultrasound has a lethal effect on the microorganisms. However, it can be used to simulate the growth of microorganisms under controlled conditions [33]. Barukčić et al., (2015) [34] have found that the application of ultrasound at 84 W for 150 s prior to fermentation promoted the activation of yoghurt culture bacteria (L.acidophilus La-5, yoghurt culture YC-380) reducing the fermentation time by 30 min compared to the control samples. Moreover, improvements in the viable cell count at the end of the fermentation was observed under thermosonication treatments (480 W at 55 °C for 8 min) which suggested the possible use of thermosonication treatment instead of pasteurisation step as it has a lethal effect on unwanted microorganisms. The collapse of bubbles create turbulence in the vicinity of the bubble collapse. This micro streaming severely damages the cell membrane of the microorganisms. In addition, the production of free radicals causes the damage to the DNA which in turn leads to cell membrane thinning [9]. The effect of ultrasound on the microbial cell membrane is known as ‘sonoporation’ which induced the formation of transient cavities or pores on the cell membrane. Figure 1 explains the sonoporation process with various interactions that are occurring between cavitating microbubbles and cell membrane [9,35,36]. Although the thickness and smoothness of the bacterial capsules play a crucial role in deactivating the microbes, positive effects are all time experienced [37].

Furthermore, the application of pulsed ultrasound, (28 kHz, 100 W/L with 100 s—on and 10 s—off pulse) for 30 min after 9 h of inoculation had a positive effect on fermentation indicating 43.5% improvement in *Lacticaseibacillus paracase* viable cell count in fermented skim milk. This is mostly due to the ultrasound-induced changes of the characteristics of cells. The changes occurred in the cell structure, or the permeability of cell membrane can positively effect on the cell proliferation [38]. Moreover, themosonication (combined application of ultrasound and heat) treatments under 25 kHz at 45 °C or 75 °C before fermentation provided honeycomb-like microstructure for yoghurts [28] and manothermosonication (combined application of ultrasound, pressure and heat) treatments under 20 kHz, 40 °C and 2 kgcm^−2^) had a positive effect on syneresis of yoghurts [32].

Reduction of fermentation time in milk samples containing *bifidobacteria* were observed under ultrasound treatment at 20 kHz [39]. When ultrasound is applied to a milk sample, it induces the rupturing of probiotic bacteria cells subsequently releasing the intracellular β-galactosidase enzyme. This enzyme promotes the hydrolysis of lactose and trans-galactosylation. The growth of the remaining bacteria cells in sonicated milk samples were promoted under the higher concentration of lactose and trans-galactosylation. Furthermore, the increase in lactose hydrolysis and transgalactosylation possibly decreased the ratio between some organic acids such as acetic acid: lactic acid, total acetic and propionic acids: lactic acid in the later stage of milk fermentation [40]. This was also aligning with the work by Sakakibara et al., (1994) [41] who experienced increased lactose hydrolysis and decreased cell viability. The presence of higher concentrations of acetic and propionic acids causes the undesirable flavours in yoghurts and therefore, use of ultrasound-assisted fermentation process contributes to improve the quality of the final product. Nobuyoshi and Etsuzo (2002) [42] found shortened fermentation times which depended on the irradiation power. With high amounts of cavitation produced increased the fermentation times. Sfakianakis et al. (2015b) [27] stated that the fermentation kinetics of ultrasound treated samples (20 kHz, amplitude150–750 W) were lower than the pressure applied samples (10–30 MPa/5 MPa) in terms of the rate of pH reduction and lag phase of pH. The possible reason for the lower lag phase of pH in sonicated samples would be the ultrasound-induced sterilization effect on milk. It could promote the onset of acidification process by providing hospitable environment for the living and growth of starter culture bacteria. However, no statistically significant differences in fermentation time were observed between the two methods. 

Contradictory, some investigations showed that application of ultrasound during yoghurt production could not be advantageous all the times. For instance, manothermosonication under 20 kHz, 2 kg pressure, and 40 °C for 12 s of thermised (60 °C/15s) cow milk prior to fermentation had a negative effect on the fermentation time though it improved the textural properties and decreased the syneresis [32]. Moreover, Wu et al., (2000) [18] found that, the application of ultrasound after inoculation contributed to significantly lower (30 min) fermentation times due to the acceleration of acid development, however, no beneficial affect was occurred on the syneresis. Furthermore, the application of ultrasound during the fermentation process adversely affected the final quality of the stirred yoghurt as the sonication process promoted the formation of lumps in stirred yoghurt [39,43,44]. This may have happened due to the formation of new bonds between proteins under low pH conditions (<5.4) and ultrasound-induced disruption of proteins may also tend to form new aggregates via newly exposed thiol groups in whey proteins [39,44]. However, several other factors were responsible for the ultrasound-assisted lumpiness of milk gels. pH: 5.4–5.1 was found as the critical pH range [44] and a dry matter content of >14.2% favourably prevented the lumpiness [43]. Sakakibara et al., (1994) [41] found that ultrasonication did not affect the ability for cell to propagate, so once ultrasonication step was finished, the count of viable cells had increased during fermentation. Therefore, it is important to carefully control the sonication parameters such as power, amplitude and time with other treatment conditions such as temperature and pressure to obtain the maximum advantages from the process. Moreover, as discussed above, the application of ultrasound before the inoculation of starter culture is more beneficial than using US after inoculation, after fermentation or during fermentation.

As discussed in previous sections ultrasound is one of the promising technologies that has been analysed for past decades to improve the properties of yoghurts. It is scientifically proved that ultrasound can be used to improve the rheological properties of yoghurts including gel strength, firmness, viscosity, water holding capacity and syneresis. Whey proteins, caseins and fat globules of milk gels are critical ingredients for the formation of milk gels and the application of ultrasound directly affect these components in a positive way improving rheological properties of the final product. 

As ultrasound is applied to milk systems, mechanical and shear forces generated within the liquid induce the denaturation of whey proteins followed by unfolding of peptide chains leading to the exposure of hydrophobic and disulphide sites. This enables unfolded whey protein peptide chains to self-aggregate or aggregate with caseins [27,28,45]. Ultrasound induced the dissociation of casein micelles into subunits and formation of aggregates with partially denatured whey proteins via disulphide interchange [28,45]. As a result, strong protein network is formed and it provides firmer gel network [46,47]. Moreover, unfolded or self-aggregated whey proteins can form micellar aggregates by interacting with κ-caseins present on the surface of casein micelles through thiol-disulphide interchange [28,45]. Furthermore, sonication induces the size reduction of fat globules and these smaller fat globules can further associate with protein aggregates providing better firmness and gel structure to the yoghurt [28]. The reduction of fat globule size increased the surface area of fat globules which thereby increase the casein bonds with milk fat globule membrane resulting better water holding capacity to the yoghurt gel. Moreover, exposure of more hydrophilic regions to the aqueous phase reduces the syneresis [18,32,48]. 

Table 1 shows the effect of ultrasound on the rheological properties of yoghurt (acid) gels. It clearly shows that, ultrasound-induced rheology improvements depend on various factors including; sonication conditions (frequency, power, amplitude, time), ultrasound application stage (before/ during/ after fermentation), composition of milk (whole milk, skim milk, whey or milk protein concentrates) and treatment conditions (pH, temperature, pressure). Therefore, it is important to carefully control these critical parameters to improve the rheological properties of yoghurt. Some investigations showed that the application of ultrasound may disadvantageous under some conditions. Longer sonication times can have an adverse effect on the water holding capacity and gel formation [39,49]. Prolonged sonication induced the dissociation of whey proteins from the formed aggregates [28] and further reduction of fat globule size results weak gel network with higher syneresis [50]. Kenari & Razavi, (2021) [51] investigated the amplitude, time and temperature on the yoghurt properties and found that hardness, viscosity and lightness increased with increase in sonication time up to 10 min, temperature up to 65 °C and amplitude up to 75%. However, increasing amplitude further decreased syneresis and flavour, while increasing temperature has led to decreased syneresis. They further found that sonication at 55 °C for 10 min at 75% amplitude was effective in obtaining a good quality yogurt. This highlights the importance of processing parameters towards the rheological benefits and how these parameters need to be manipulated to obtain a good quality yoghurt. 

The application of ultrasound to milk can cause several alterations to the chemical structures of the components in milk leading significant changes to the sensory and nutritional properties of milk or resulted secondary milk-based products. The ultrasound-induced cavitation process and the generation of localized extremely high temperature and pressure gradients result chemical reactions which can lead to the formation of free radicals and some reactive species. For instance, decomposition of water generates H+ and OH- consequently creating reactive oxygen species (ROS) such as H_2_O_2_ during the recombination process. Moreover, ultrasound may induce the formation of volatile molecules which can deteriorate the flavour and aroma of milk. This process is stimulated by the formed reactive oxygen species as their transferring process between the gas bubbles and liquid phase induce the redox reaction with the solutes in milk [4,59,60]. A recent study by Bui et al. (2021) [61] found that short sonication times (<5 min) did not significantly change the volatile profile of milk, which in contrast showed the production of volatile compounds and changes in the amounts of fatty acids significantly changed with prolonged sonication times (~10 min). Therefore, the influence of ultrasound on the sensory and nutritional properties of the milk-based products like yoghurt is very important as the consumer acceptance significantly depends on the flavour, aroma, taste and colour of the product. As the production of yoghurt involves several steps, each step may affect the sensory attributes of the final product while the fermentation is the most influencing step. During fermentation, flavour generating products such as volatile acids (butyric and acetic), non-volatile acids (lactic and pyruvic), carbonyl compounds (acetaldehyde and 2,3-butadione) and miscellaneous compounds can be formed [62].

Yoghurt prepared from sonicated milk (20 kHz frequency, 150–750 W power levels for 10 min) showed the lower degree of likeness and off-flavours while having higher burned, pungent and fatty flavour attributes compared to the pressure treated (first stage: 10–30 MPa and second stage 5 MPa) yoghurt [63]. Moreover, increase in the concentration of long-chain aldehyde molecules and several ketones were also observed in ultrasound-treated samples. Because of the modification of flavour-related compounds, ultrasound-treated samples had sweeter and more acidic flavours with less acceptability. Interestingly, yoghurt prepared under ultrasound power of 150 W and pressure treated yoghurts had similar flavour and taste profiles while 375 W power resulted the most negative impact on the sensory qualities denoting increase in ultrasound power caused to the detrimental effect. The volatiles produced during sonication belonged to the groups of aldehydes, ketones, esters, alcohols and hydrocarbons and were the products of oxidation of lipids or protein degradation due to acoustic cavitation [61].

The comparison between yoghurt prepared from thermosonicated milk (24 kHz frequency at 45 °C for 10 min) and conventional heat treated milk (90 °C for 10 min) showed significant differences in colour, sensory attributes and vitamin retention between samples [52]. In terms of colour properties, yoghurt prepared after ultrasonication treatment had slightly higher L* (lightness) values and significantly lower a* (redness/greenness) compared to the heat treated samples and it implies that ultrasound had the less thermal impact and reduced the tendency to nonenzymatic browning compared to the heat treated samples [52]. It is shown that the overall acceptability of thermosonicated milk is higher in the yoghurt samples prepared with 0.1% fat content than the heat-treated samples. For milk with 1.5% of fat, sensory differences were less distinct between samples. Another important finding of this research work is the analysis of vitamin retention of sonicated samples. According to the observations, thermal liability of vitamins; thiamine, riboflavin, a-tocopherol and retinol were not negatively affected by either thermosonication or heat treatments [52]. In contrast, another study showed that, yoghurt prepared from thermosonicated sweet whey under the condition of 480 W at 55 °C for 8 min obtained the lowest score in sensory analysis due to the strong acidic taste of the samples. However, the colour of the yoghurt was brighter in thermosonicated samples as there were no sediment formation occurred after the fermentation process. The possible reasons for this effect could be the ultrasound-induced protein solubility in whey samples [34].

### 4.2. HPP Applications in Yoghurt Products

The use of HPP is a useful non-thermal technology and it has unique features to modify a few physico-chemical and functional qualities of processed products including yogurts and other yogurt based products to give positive attributes. Low firmness, syneresis and viscosity are the most common undesirable features of both set and stirred yogurts [64]. However, the application of HPP during the production of yoghurt and yoghurt products have showed several beneficial effects in order to overcome the above mentioned issues and to improve the process and product quality of yoghurt-based products in several ways.

Considering the rheological properties of yoghurts, improved qualities in firmness, the resistance to fracture and syneresis and improved viscosity were observed in yoghurts prepared from HP-treated milk [65]. Skim milk treated with a combination of 400 to 500 MPa of HPP and 85 C heat treatment for 30 min showed increased yield stress, resistance to normal penetration, elastic modulus, and reduced syneresis [66]. Moreover, Set yoghurt produced at 60 MPa for a process time of 15 min in comparison to thermally processed showed lower fracture stress, according to Needs et al. (2000) [67]. Similarly, after centrifuging for 25 min, the acid gels made from HPP milk displayed a linear decline in whey holding capacity retaining 20% of whey [68]. Another research work on low- fat yoghurt prepared using 676 MPa for 5 min at room temperature conditions had more interconnected chains in its dense aggregated protein structure, a smoother surface, and a compact gel with enhanced gel texture and viscosity than the untreated yoghurt samples [69]. Because of the modifications in the gel structure and water-binding abilities of proteins, yoghurt prepared from milk that had undergone high pressure conditions was less vulnerable to undergo unwanted syneresis after storage [70]. High pressure processing at elevated temperatures would result in a reduction in the final viscosity of yoghurt, and is noted in the 100–400 MPa and 90 °C processed according to research on the production of stirred yoghurt [71].

In yoghurts, Serra, Trujillo, Guamis, and Ferragut (2009a) [72] studied the proteolysis of two milk samples, one sample is made using 200 and 300 MPa HPP at two different temperatures such a 30 and 40 °C and it was compared against heat-treated milk containing 3 percent skim milk powder. Only HPP milk that was processed at 300 MPa produced yoghurts with comparable proteolytic characteristics of heat-treated milk containing 3 percent skim milk powder. Additionally, the authors discovered hydrophobic peptides at detectable amounts, which is particularly interesting given their possible biological actions. Even when no skim milk powder was added to the HPP treated samples, these peptides showed comparable amounts in yoghurt made from ordinary milk and yoghurt made from HPP milk treated at 300 MPa. Due to the improved quality characteristics, such as less syneresis and higher gel stiffness than yoghurts manufactured from heat-treated milk combined with 3 percent skim milk powder, the 300 MPa was therefore considered the best condition for yoghurt manufacturing.

Since yogurt is one of the mostly consumed probiotic product throughout the world, HPP should not negatively affect on the viability and persistence of the probiotic strains during the product life cycle as pro-biotic bacteria need to be alive in satisfactory levels to make a positive impact on consumers’ health [73]. A relatively longer shelf-life probiotic yoghurt was produced between 350 and 650 MPa and process temperature of 10 to 15 °C. Spoilage microorganisms like yeasts and molds were inactivated in this method but not specially selected pressure-resistant probiotics, extending the shelf life of yogurt to 90 days [74].

A method for extending the shelf life of probiotic yoghurt using high pressure treatment to kill off spoilage microorganisms while keeping specifically chosen baro-resistant types of probiotic bacteria viable has been patented by Fonterra’s company in New Zealand [75]. They have also explored the use of HPP for the preservation of colostrum and increased shelf-life for yogurts [76]. The flavour profiles and survivability of starter cultures of yoghurt made using milk processed at 200 or 300 MPa by HPP at temperature of 30 or 40 °C were assessed by Serra, Trujillo, Guamis, & Ferragut (2009a) [72]. *Streptococcus thermophilus* was found to be more prevalent than *Lactobacillus delbrueckii* spp. bulgaricus in all products until storage, and yoghurts manufactured from heat-treated skim milk had a greater total cell load than yoghurts made from milk processed by HPP. The scientists also noted higher levels of organic acids in yoghurts manufactured from milk processed by HPP at the specified temperatures. Yogurts made from heated milk displayed less connected micelles and uneven, structures with large-pores. Contrarily, yoghurts produced with milk processed at high pressure and heat along with high pressure treatment shown little syneresis, whether *streptococci* or *lactobacilli* strains were utilised. According to the scientists, probiotic yoghurts from milk obtained using high pressure process produced gels with higher consistency indices, and the yogurt’s gel firmness depended on the starter culture used [77]. Conclusively, a creamy and thick consistent yogurt was observed that require no addition of stabilizers from the combined HP and heat-treated milk. More importantly these treatments did not change the properties and microbial counts of both the starter and probiotics [69,78].

The post- acidification which occurs during the storage of fermented products like yogurts which is known as the continuous pH decrease is a major problem occurs in dairy industry. This phenomenon can be called as a reduction of pH values after fermentation and during refrigeration, mainly because of the uncontrollable growth of *L. delbrueckii ssp bulgaricus* in these conditions [79]. This affects the sensorial properties and the shelf life of yogurt which is noticeable to consumers, which can cause the product rejections [80]. Regarding this matter, Ancos, Pilar Cano, and Go’mez (2000) [81] discovered that Lactobacillus bugaricus was more sensitive than S. thermophilus under HPP treatments when milk was processed by high pressure between 100–400 MPa for the production of skim beaten yoghurt. Additionally, it showed a decrease at 300 and 400 MPa that ranged between 1.9 and 4.0 log UFC/g. Only 0.1 pH points were lost as a result of this lowering after 20 days in the refrigerator conditions. This is crucial for the survival of probiotic microorganisms, particularly Bifidobacteria species, which are more vulnerable to low pH levels.

The popularity of HPP arises from its ability to process foods with fresh-like taste with no chemical preservatives with extended shelf-life [82]. In a study to evaluate the range of modifications occurring during combined heat and HPP treatment in yoghurt manufacturing, Harte et al., 2003 [78] examined the impact of HPP on colour. They found that milk that has undergone heat treatment followed by HHP sheds its white colour and becomes yellow, whereas milk that has undergone HPP first and then thermal treatment has regained its white colour. The first observation may be caused by a reduction in casein micelle size, while the second may be caused by the reversible nature of casein micelles when subjected to HPP treatment in between 300 and 676 MPa before the heat treatment. With regard to increasing yogurt consistency, high intensities must be used and it is done via four main steps. They are; (1) breakdown of casein micelles physically (2) limited amount of colloidal calcium phosphate dissolution, (3) b-lactoglobulin denaturation caused by higher pressure upon longer exposure time, (4) Achieving more protein interactions by casein modifications. Therefore, through these consequences more consistent yoghurt products can be achieved [83].

By destroying the cell membrane and rupturing non-covalent linkages, HPP renders vegetative microbes inactive. High pressure changes the structural and functional stability of macromolecules like proteins and polysaccharides by disrupting their secondary and tertiary structures in a pressure-dependent manner [84]. Gram-positive microorganisms are more resistant to pressure than Gram-negative bacteria and it was explained that Gram-positive organisms need an application of 500–600 MPa at 25 °C for 10 min to be inactivated while Gram- negative organisms need only 300–400 MPa [85]. HPP treatment is also known to inactivate milk microorganisms logarithmically such as; *L. monocytogenes* in milk at 375 MPa and *S. aureus* at 600 MPa depending on the size, shape of bacteria and also the nature of cell membrane [86]. For the inactivation of spores, HPP treatment at pressures above 600 MPa is combined with heat treatment (60–90 °C) and the spore germination is increased under pressure of 400 MPa leading to vegetative cells inactivation [87]. Therefore, HPP can be positively adapted to manufacture milk with a extended shelf life with no unwanted flavor which is a common feature of high temperature processing. Anyhow, when processing skim milk, since it becomes semi-transparent after being processed at pressures above 400 MPa it can be a limitation [88]. Another study says that samples of ayran yogurt drink treated at 600 MPa during 5 min had reduced in the levels of *L. monocytogenes* and *L. innocua* by more than five log units (*p* < 0.05) at ambient temperature [89].

Regarding the HPP effect on proteins, it effects on dairy proteins including size reduction of micelles, denaturation of whey protein, increased solubility of calcium and colour change [84]. Moreover, the homogenization pressure could encourage some conformational changes in whey proteins and caseins, increasing their susceptibility to proteolysis. This helps to increase the availability of free amino acids, which are used by probiotic bacteria directly [90]. Along with that whey proteins, which are structurally stable and not interactable with caseins, fat globules or calcium ions in their native form get denatured after homogenization and bind with fat particles and caseins giving important properties for yogurt production [86].

L. Parekh, Aparnathi and Sreeja, 2017 [91] and Walker et al., 2017 [92] have described the usage of HPP for fruit yogurts. 550 MPa was used to produce the samples, which were then kept at 20 °C or 4 °C for 4 weeks. The quantity of bacteria in the high pressure treated yoghurt held at 4 °C was <10^6^ CFU/mL and moreover pressure administration prevented the post acidification of the product, according to their findings. Furthermore, they observed that even 60 days of storage at refrigerated and room temperature had not resulted in any microbial deterioration in the high pressure sample. The number of LAB bacteria also dropped to <10 CFU/mL.

As a conclusion, despite all the downsides, and all food processing and packaging encountered challenges, HPP has demonstrated sufficient benefits of minimal alteration of the yoghurt products and process/product efficiencies that can be a more significant food preservation force in the future.

### 4.3. Encapsulation of Nutraceuticals into Yoghurt Products Using US and HPP Technologies

Encapsulation is known as a common process used in food industry to cover various substances in matrices of liquid, gas/gaseous, and solid [93]. Lipids, proteins, carbohydrates which have important properties like a low hygroscopy, high solubility, low viscosity, film-forming capacity, low cost, and the capability of producing a highly stable emulsion are used as the most frequent wall materials/covering matrices in encapsulation systems [94]. Therefore, dairy products like yoghurt, cheese, ice cream, fruits and vegetable juices and fermented beverages can all be functionalized by encapsulation with different beneficial bio actives such as essential oils, vegetable oils, fish oils, and plant extracts in the food industry [95]. Also, there are various applicable micro/nanoencapsulation methods such as ultrasonication and high-pressure processing for encapsulation of which can facilitate their use in the various food formulations [96].

A number of studies based on the encapsulation of nutraceuticals in yogurt by ultrasonication have highlighted the fact that it develops the textural parameters, improves functional properties and extends the shelf life by maintaining acidity [97]. The effectiveness and total acceptability of additives in yogurt is decreased by many factors during processing, due to degradation, oxidation, and undesirable reactions with milk proteins [98]. In order to enhance the antioxidant activity of yoghurt, a study was carried out by the encapsulation of powdered Doum extract in liposomes by ultrasonication [99]. The encapsulation of active ingredients in liposomes can advance their bioavailability by protecting them against oxygen, acids, and processing conditions. Moreover, it has the ability to disperse lipid compounds into an aqueous phase for improved delivery and control of release [100]. The results indicated that the Doum extract powder (DEP) can be successfully encapsulated in liposomes and the high encapsulation efficiency, particle size, and TEM examination indicate successful encapsulation of up to 1% of extract powder. Yogurts with the addition of 5% Doum extract powder liposomes, had some favorable effects on the development of acidity, textural parameters, and water holding capacity, compared to the control. The addition of higher percentages of DEP liposomes can significantly affected the functional properties of yoghurt. Therefore, in order to increase antioxidant activity in yogurts, 5% DEP was recommended.

Another aspect of this technology is encapsulation of dairy starters along with probiotics into yogurts. This has two advantages: it reduces cell damage from unfavourable conditions such reactive oxygen, organic acids and hydrogen peroxide; and it slows down the encapsulated cell’s rate of multiplication, substantially limiting post-acidification [101]. According to Oh et al., 2006 [102] yoghurt with microencapsulated crude bacteriocin demonstrated significant pH differences against control as 4.37 and 3.92, respectively, after 24 h of fermentation at 42 °C and with barely any pH rise after 20 days of storage at room temperature. *Bifidobacterium breve* has been encapsulated in whey protein microcapsules [103] and alginate-goat’s milk inulin probiotic encapsulate for goat’s milk yoghurt [104] are two other studies on a related issue. The immobilization of *bifidobacteria* in water-insoluble whey protein-based microcapsules can increase their tolerance to high acid environments, making this approach potentially useful for delivery of probiotic cultures to the gastro-intestinal tract of humans. However, these findings highlight the need to take into consideration the technological properties of probiotic strains with regard to processing, and particularly heat stability to stabilize sensitive cultures. Future work should involve investigations of techniques which could prevent cell damage and optimize viability during the process, such as stress adaptation during cell preparation. On the other hand, the results of alginate-goats’s milk inulin probiotic encapsulation showed that this matrix has potential to be used as a new encapsulation material to encapsulate probiotics for use in goats’ milk-based probiotic fermented dairy products, avoiding the cross-contamination caused by using capsules based on cows’ milk. However, a sensory evaluation was suggested to be conducted in order to have a clear idea about how the capsules affect the sensory properties of the probiotic goats’ milk yoghurt, such as colour, texture, acidity and flavour.

Another study on evaluating the potential uses of microencapsulated rice bran oil (RBO) as a bioactive compound and utilizing it in preparing yoghurts, indicated that there were changes in values of organoleptic parameters of control and different tested levels of supplemented yoghurts with RBO and encapsulated RBO during the storage periods of 7 and 14 days. RBO was encapsulated with wall materials such as maltodextrin (MD): Whey protein concentrate (WPC), MD:WPC: gum arabic (GA) and MD:GA at ratios (3:2), (3:1:1) and (3:2). Use of MD: GA as a wall material with core to coat material at ratios 1:4 for encapsulation of RBO caused a decreased in the surface oil content (0.22) and an increase in encapsulation efficiency to 78%. Adding powder of encapsulated RBO at different concentrations (2, 4 and 6%) and RBO at 2% to yoghurt samples caused a decrease in pH and increase in values of acidity and water holding capacity (WHC) during the storage period. This may be due to the increase of the amount of bound water and decrease in whey separation. It was reported that low WHC and whey separation are related to an unstable gel network and extreme rearrangement of a weak gel network. According to the results obtained, these changes were still within the acceptable scores and satisfaction of sensory panelists [105].

Moreover, milk which is the main ingredient of yogurt, has been broadly engaged in creating natural casein micelle nano capsules with high nanoencapsulation efficiency with the use of alkaline pH and ultrasonication. This sustains their natural structure and morphological characteristics due to electrostatic repulsions that facilitate interior hydrophobic areas. This is a great benefit in the encapsulation systems of unsaturated fatty acids, oils, and other hydrophobic compounds used to enrich and fortify food as well as pharmaceutical products. Abbasi et al., (2011) [106] conducted a research on the capability of ultrasound for encapsulation of ω-3 fatty acids in order to produce ω-3 enriched Doogh which is an Iranian yoghurt drink. As per the findings, sonication at different amplitudes and exposure times was able to encapsulate as well as homogenize omega-3 fatty acids in enriched Doogh system. Furthermore, the samples showed excellent physical stability during storage period. Breaking down the oil droplets to smaller globules and the proteins in yogurts by sonication energy could coat the globules and lead to the absence of oil on the surface of Doogh. Moreover, the presence of stabilizer possibly, due to electrostatic and steric repulsions, interacted between the oil globules encapsulated by proteins and physical stability extended.

**Figure 2:**
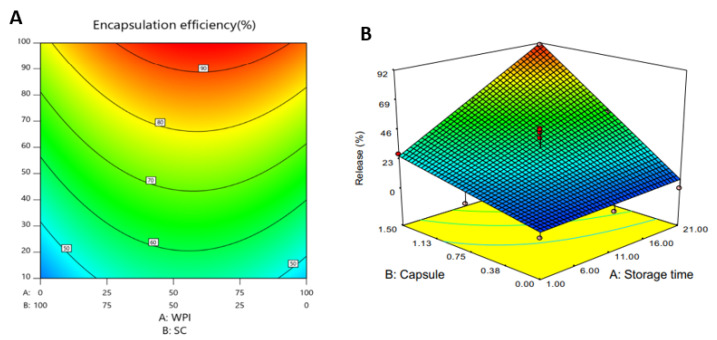
(**A**) Contour plots of encapsulation efficiency against sodium caseinate/isolated whey protein, ultrasound power and core to wall ration and (**B**) Three-dimensional curve of effect of storage time and microcapsule content in yoghurt on release rate of essential oil from microcapsules. Reproduced from [108] with permission from John Wiley and Sons, 2022.

In order to demonstrate the nano emulsion delivery systems in enhancing omega-3 fatty acids absorption in yogurts, soy lecithin in combination with 50% DHA algae oil and water was conducted under the ultrasound emulsification method. The resulting nanoemulsion was then mixed with strawberry yoghurt and the size of nanoemulsion was 258 nm. In comparison to bulk oil-enriched yoghurt, the results demonstrated that nanoemulsion technology enhanced the PUFA’s bioavailability [107]. In research, where flavored yogurt was prepared by the encapsulation of Melissa officinalis essential oil through ultrasonication, using different ratios of whey protein isolate/sodium caseinate as coating material the release behaviour of the essential oil was characterized over a 21-day storage period (Figure 2). After the quantified results by dispersive liquid–liquid microextraction followed by gas chromatography, it was identified that the antioxidative activity of yoghurt samples was increased by the incorporation of encapsulated essential oil. Therefore, the use of microcapsules containing M. officinalis essential oil could be a suitable method for producing nutraceutical foods with antioxidant properties in dairy industry [108].

Some research recommends co-encapsulation with prebiotics, for increasing the probiotics activity and survivability. It has been observed that encapsulation of Lactobacillus and Bifidobacterium species in yoghurt by fructooligosaccharides improves bacterial count and does not have any undesirable effect on chemical properties [109]. Furthermore, a recent study which was implemented on the potential use of ultrasonic emulsification process for encapsulation and delivery of nutritional compounds in dairy-based drinks has summarized, viscosity, improved heat stability, reduced syneresis, increased gel strength and delivery of high value nutrients as the key functionalities could be achieved by ultrasonic processing of food products in dairy systems. Anyhow the study recommends to develop appropriate large scale food processing equipment is lacking along with the successfully carried out pilot studies in the dairy industry [110].

On the other hand, when considering the encapsulation of nutraceuticals into yogurt by high pressure processing given the potential of HPP in controlling post-acidification, and improving the organoleptic properties of yogurts, an emerging need of studies pertaining for the encapsulation by HPP in yogurts can be identified due to the lack of research. HPP is also a high-energy method, such as sonication to generate powerful disruptive force to form small oil droplets by high shear stress, while the mixture of oil and water phases is pumped through the restrictive valve of a high-pressure. The main factors that influence the size of droplets and the properties of nanoemulsions are the amount of energy applied and the choice of surfactants and other additives [107].

Although the research related to encapsulation in yogurts by HPP is minimal, recently, Golfomitsou et al. (2018) [111] studied oil-in-water edible nanoemulsions as carrier of vitamin D in order to fortify dairy emulsions. The emulsifiers used for the nanoemulsions were polysorbate and soybean lecithin and the nanoemulsions were prepared by using a high-pressure homogenizer to obtain at the end a product containing oil droplets with mean particle diameter less than 200 nm. The results showed that the droplet diameter of the milk emulsion was not influenced by the presence of the loaded nanoemulsion and the milk which was fortified was stable in regard of particle size and gravitational separation for minimum 10 days. Finally, the vitamin which was reported to have radical scavenging activity assessed by electron paramagnetic resonance (EPR). Another study on encapsulation of *Clitoria ternatea* extract in liposomes by synergistic combination of probe-type ultrasonication and high-pressure processing suggested that HPP at pressure ranging from 300 to 600 MPa are suitable to be applied in the dairy beverage, pharmaceutical, and cosmetic industries mainly to reduce particle size of liposome and consequently increase uniform of distribution [112]. Moreover, the results showed that the HPP had the lowest influence on particle size in the preparation of the encapsulated *Clitoria ternatea* when compared to the other homogenization modes like ultrasonic bath, probe-type ultrasonication, and magnet stirring. Anyhow the encapsulation efficiency of liposome by HPP was still >70% for *Clitoria ternatea* petal extract. Moreover, the particle size of liposome was effectively decreased with the increase of pressure via the pre-processing of probe-sonication and post-processing of HPP [113].

Futuristically, emerging technologies like ultrasonication and HPP need further explorations and regulatory approval for application in food products. Specifically, when talking about the yogurt manufacturing, these processing techniques being, environment friendly, sustainable and innovative can gain momentum in the encapsulation of bio actives as well as probiotics. Anyhow the use of expensive encapsulant material will add cost from one end while it can affect the sensory attributes of products on the other. Therefore, careful research, pilot and feasibility studies, machinery development and legislations are needed to get the optimum use of these novel approaches.

## 5. Beverage Products

Beverages play a vital role in human diets from newborn to the elderly. Any fluid designed or developed for human consumption by drinking can be called as a beverage. There are four primary sectors of beverages globally which are; hot drinks, soft drinks, alcoholic beverages and milk drinks [114]. In another approach beverages are classified under five various criteria as natural and synthetic based on the ingredients used in manufacturing, carbonated and non-carbonated based on the degree of mechanical carbonation, alcoholic and non-alcoholic based on the presence of presence of alcohol, hot and cold based on the serving temperature and stimulating and non-stimulating based on the physiological effect [112]. Although beverages are not consumed for its food value occasionally, their prime function is hydration. Therefore, beverages are available from polar bases to the tropics, throughout the globe from in bottles, cans, laminated paper packs, pouches, cups and almost every other form of packaging known and manufactured under different processing conditions which are unique to each beverage type. Moreover, application of novel technologies in the beverage industry is an emerging need because of its fast-growing nature.

### 5.1. US Applications in Beverage Products

The primary application of ultrasound in dairy beverage processing is to inactivate microbes and enzymes without altering the taste and nutrients and improving the physical and structural properties and used an alternative for traditional pasteurization treatment [115]. Ultrasound sometimes has a unique application for the distribution and size reduction of particles to obtain the stable dairy beverage, some studies have said about the improvement in the fermentation process, increase in the content of oligosaccharides and bioactive peptides, and the lowering of the range of lactose in some fermented dairy beverages [116,117].

The ultrasound can destroy various microbes, which can act as the non-thermal pasteurization of dairy beverages. It’s capable of reduction of aerobic mesophilic microorganisms with log reduction of 3.56 ± 0.02, aerobic mesophilic heterotrophic bacteria count with log reduction of 0.03 ± 0.09, molds and yeast with log reduction of 0.2 ± 0.2 at 200 W [118,119]. In dairy beverages, the addition of external ingredients is added for flavour, color, appearance, or health benefits, i.e., functional oils needed to be adequately solubilized or homogenized in beverage to obtain fine quality out by ultrasound process effectively [116]. Monteiro et al. (2018) [119] used high intensity ultrasound (HIUS) in two-level HIUS-A (160 W for 937 s) and HIUS-B (720 W for 208 s) to prepare chocolate whey beverages. They found a better fat size globule reduction for HIUS-B and was 0.5 µm from 10 µm, than HIUS-A, which was 1 µm from 300 µm Both treatments showed preservation of minerals in a beverage.

Monteiro et al. (2018) [119] worked on ultrasound at 19 kHz, 400 W power, and energy density 0.3, 0.9, 1.8, 2.4, and 3.0 kJ/cm^3^ found that HIUS led to good fat size distribution and high homogenization efficiency. In the same work, authors have also found that 29 volatile compounds were present in both HIUS treated and untreated samples and the amount of antioxidants present after application of energy density 3.0 kJ/cm^3^ and 0.9 kJ/cm^3^ were 87.2 ± 0.5% and 88.2 ± 1.3%. However, there was no effect on polyunsaturated fatty acids and monounsaturated fatty acids and allowed better preservation of short-chain medium-chain fatty acids. The energy density of 3.0 kJ/cm^3^ reduced total aerobic counts to 3.56 ± 0.02 logarithmic cycles. In another work, (Guimarães et al., 2018) [118] prepared prebiotic inulin enriched whey beverage using ultrasound at 19 kHz power and 0,200,400,600 W for 3 min. It showed similar microbial inactivation as thermal HTST (72 °C for 15 s). Also, ultrasound 400 and 600 W showed final microbial counts of less than the limit of detection (1 log CFU g^−1^), reduced viscosity which is attributed to the reduction in the size of casein micelles and a good consistency due to cavitation effect resulting in breakdown of the particles in the medium. It also improved the kinetic stability, disruption of milk and fruit cell and these changes are attributed to the decrease in particle size and the interaction between molecules because of the physical effects of ultrasound.

Likewise, the application of the ultrasound in modified whey protein suspension has shown smaller particle size formation, higher solubility, and thermal stability. The improved thermal stability reduced the constraint associated with whey protein during thermal processing when thermal processing was carried out after ultrasonication, and the whey protein can withstand higher temperatures during processing for a longer time due to breakdown of polypeptides chain and because of the deactivated the free–SH groups which ultimately reduced the protein re-aggregation [120]. The ultrasound in prebiotic and probiotic dairy beverage products has both acceleration or inhibition effect on the cells [121]. The ultrasound can produce sonoporation, i.e., the formation of pores in the cell membrane. Low energy improves the mass transfer and supply of nutrients, and high energy can cause breaking of the lipid membrane, causing cell damage [36]. Ultrasound is also applied to milk and other milk products that have gained importance recently, such as fermented milk products, ice creams, butter, etc. [122,123]. The ultrasound may be used for specific or combined effects such as defoaming, emulsification, ice crystallization, lipid crystallization, or changing the fermentation time [124]. All have a beneficial effect on milk properties, and similar studies can be applied to dairy beverages.

In a summary, US processing of different beverages has generated a great interest in the aspects of enhanced emulsification, improved homogenization, retention of quality parameters, increased stability and reduced spoilage. However certain aspects of US treatment like US duration and fruit juice extraction yields, the availability of US treatment in industrial scale or pilot scale especially related to the dairy industry and the complex physical-chemical mechanisms underlying the actions of US and its effects on beverages should further studied to strengthen the future applications of US treatment in beverage industry.

### 5.2. HPP Applications in Beverage Products

Growing consumer demand for high-quality beverages has driven adoption of non-thermal technologies such as high-pressure processing (HPP) in the beverage industry, because they perfectly adapt, unlike the traditional thermal and chemical treatments.

Bi et al. (2020) [125] studied the effect of HPP on the mango smoothie at 500 MPa/8 min and 600 MPa/5 min, and compared with heat treatment at 90 °C/20 min. They found that reductions of 3.53 log10 cycles and 3.95 log10 cycles were achieved by treatment at 500 MPa/8 min and 600 MPa/5 min, respectively. Also found that, the total plate count and yeast and mold (Y&M) count decreased rapidly at initial 0–2 min due to organism which are sensitive to pressure effect and declined at a slower rate at 2–15 min due to pressure resistance microbes. During storage for a period of 15 days, yeast and mold counts were lower than 1 log10 CFU mL^−1^. This is for the reason that the cell wall of yeast and mold were more sensitive to HPP as compared to total plate count due to their inherent cell structure. Similarly, the viscosity of mango smoothies treated with heat for 500 MPa/8 min and 600 MPa/5 min increased by 94.1%, 32.6%, and 107.7%, respectively. Due to HPP treatment the concentration of ionic calcium in milk rises and pectin in smoothies reacts with the Ca^2+^ ions present to form calcium pectate and other compounds causing the increase of viscosity while in heat treated milk a combine effect of rise in volume fraction and increased interaction between casein micelles causes the increase of viscosity of smoothies. However, the reaction between pectin and calcium reduces as there is degradation of pectin as pressure increases, which is also responsible for reduction in the turbidity at higher pressures. But no significant difference was observed in the pH of samples.

Pega et al. (2018) [126] used HPP at 200 MPa/10 min and 400 MPa/1 min on sweet whey fermented beverages and compared the results to control samples (without treatment) using lactic acid bacteria starter to evaluate the impact after post acidification. The plate count for starter lactic acid bacteria was lower in the HPP treated sample than in the control sample. Still, treatment at 200 MPa maintained the optimal amount of starter lactic acid bacteria 6.6-7.9 logs CFU/mL, treatment at 400 MPa starter lactic acid bacteria was reduced by nearly 5 log reduction was observed after 30days and maintained at 3.0 logs CFU/mL up to 45 days. There was no colour difference, but after 45 days, the sensory score indicated HPP at 200 MPa was better than 400 MPa, but there was no significant difference, while the control sample showed a larger significant difference for change in flavour and texture.

In a similar study, Bansal et al. (2019) [127] checked the effect of HPP (500 MPa/10 min at 25 °C) on the sweet lime whey beverage and compared it against the heat treatment at 90 °C/60 s. They found that HPP was able to keep a count of mesophiles, yeast, and coliforms to less than 1 log CFU/mL for 120 days at 4°C from the initial count of 8 log CFU/mL in the untreated sample. Higher shelf life was due to the reduction in pH that favoured the inactivation of microbial load and the antimicrobial property of bioactive compounds in beverage, and at the same time, the heat-treated sample kept a count less than 1.2 log CFU/mL. The total phenolic compound in fresh whey lime beverage was 549 ± 0.3 mg GAE L^−1^ (mg of gallic acid equivalents per liter), and after heat treatment, it was only 8.6% (502 ± 0.13 mg GAE L^−1^) and was 62.1% (208 ± 0.21 mg GAE L^−1^) after 120 days. But just after treating with HPP, it was increased by 1.6% (~558 ± 0.3 mg GAE L^−1^) and retained upto 60.2% (329 ± 0.19 mg GAE L^−1^) for 120 days. The increase in total phenolics upon HPP was due to the release of phenolic compounds under high pressure treatment.

In another study, Zhang et al. (2020) [128] checked the effect t of HPP on egg white whey protein mixture at acidic condition of pH 3.5 with the purpose of controlling the astringency of whey protein. The samples were treated with HPP at 450 MPa and 600 MPa for 3.5 min and compared against same formulation with thermally denatured protein treatment and conditions. The turbidity and absorbance of the samples were increasing by increasing the addition of egg white to whey due to the formation of gel structure in case of thermally denatured protein treatment however no significant difference was noted among the HPP treatments. But the astringency increased with HPP treatment due to interactions between the whey and salivary proteins. In a different study, Diez-Sánchez et al. (2020) [129] prepared milkshakes using chokeberry pomace with the application of HPP treatment at 500 MPa for 10 min. The 10% chokeberry pomace showed better retention of antioxidant capacity and phenolic compounds with minimum microbial survival. However, antimicrobial properties of the pomace was partially camouflaged by interactions between milk proteins and polyphenols, and by the longer HPP treatments which in turn led to higher level of interaction.

In a recent study by Munir et al. (2020) [90] HPP at 400 MPa was used as a pre-treatment technique for milk in the preparation of cheddar cheese for a treatment time of 15 min. They found that HPP treated cheese milk showed increase in ACE Inhibitory activity due to changes caused in the structure of milk enzymes that would react with the proteins ultimately resulting in efficient proteolysis and release of bioactive peptides. Higher antioxidant activity and DPPH radical scavenging activity were noted in HPP processed cheese milk and was possibly due to the pressure-induced changes in casein structure, making the proteins more reactive to enzymes.

There are some application for raw milk (Stratakos et al., 2019) [130] applied 400, 500, and 600 MPa hold time of 1, 3, and 5 min respectively HPP made 5 log reductions for pathogenic microbes like *E. coli*, *Salmonella* and *L. monocytogenes* HPP milk had similar casein and fat particle sizes compared to pasteurized milk. While (Liepa et al., 2018) [131]. Applied 250 MPa/15 min; 400 MPa/3 min; 400 MPa/15 min; 550 MPa/3 min for skimmed milk and found that, minimum 400 MPa and 15 min treatment is required for shelf-life extension of skimmed milk 550 MPa for 3 min the total count of microorganisms was stable at 1.53 log CFU/mL.

Conclusively, HPP technology can be allowed in obtaining beverages with improved nutritional and functional qualities like flavour, color, texture, viscosity with a longer shelf life due to the inactivation of microorganisms. Anyhow the applicability of HPP treatment on beverages should be widely studied to fulfill the gaps and understand the process completely to reduce the cost of production.

### 5.3. Encapsulation of Nutraceuticals into Beverage Products Using US and HPP Technologies

A study by Shanmugam & Ashokkumar, 2014a [132], reported stable delivery of nutraceutical, the flaxseed oil of about 7 and 21% in skim milk using 20 kHz US and 3 and 6 min of sonication time at 176 W for up to 9 days at 4 ± 2 °C. The particle size of low oil of 7% and high oil of 21% emulsion were 0.64 and 0.85 μm, respectively. The flaxseed oil has 59.9% of a-linolenic acid (ALA), an omega-3 fatty acid in it. ALA is an important fatty acid that has been shown to help children’s cell, nerve, and cognitive development, as well as human cardiovascular functions. The generation of smaller emulsion droplets and process-induced changes of milk proteins are due to the mechanical, cavitational, and cavitation-after-effects of US. The emulsion droplets were stabilised by a tiny percentage of partially denatured whey proteins (less than 20%) [28]. Due to the adsorbed partially denatured whey proteins, the emulsion droplets had a surface potential of roughly -30 mV, which provided additional stability to the emulsion droplets due to electrostatic repulsion. Experiments were carried out using Ultraturrax (UT) at similar energy densities as US to explore if other high shear techniques may yield stable emulsions. UT did not create stable emulsions until 20 min after processing, indicating that the US emulsification procedure is superior [132]. In a similar study, milk beverage enriched with emulsions of black cumin seed oil (Nigella sativa L.) was produced using US at 20 kHz at 100 W at a process time of 8 min with droplet size of 0.4 μm. The black seed oil is known to produce healthier lipid profile and blood pressure [45]. Another study on carrot juice beverage created stable emulsions of 1% flaxseed oil utilising a 20-kHz ultrasound (US) at 176 W in 4 min. The creation of emulsion droplets with a diameter of 0.6 μm is due to the shear forces created by sonic cavitation. Pectin worked as an emulsifier, causing electrostatic repulsion (potential 30 mV) between emulsion droplets, which kept the emulsions stable for up to 8 days at 4 ± 2 °C. The emulsion’s viscosity was unaffected by sonication period (up to 8 min) or storage. The beverage emulsions’ undisturbed flow characteristics made it easier to adapt to existing pipelines in the food industry [133]. The use of the US approach to create stable emulsions without addition of external emulsifiers and stabilisers or at larger quantities is considered as vital in the creation of novel products and processes. Consumer desire for healthy food items is one of the most important developments in the food industry. Encapsulated bioactives are one of the most widely used strategies for producing functional foods. According to the most recent WHO reports, vitamin B12 deficiency will become widespread over the world, and it will likely become the most common malnutrition in the future [134]. B12 is an essential co-enzyme for cell growth, and a lack of it in the body can cause weakness, diarrhoea, weight loss, anaemia, and weariness [135]. As a result, the capacity of US in producing double emulsions of W1/O/W2 emulsions after making primary emulsion of W1/O to deliver 0.2% vitamin B12 into skim milk was studied by Zaghian & Goli, 2020 [136]. For better stability of double emulsions, the nano sized stable emulsion droplets of primary emulsion is important and is obtained at 10–12% of W1/O (20% of W1 of skim milk in 80% sunflower oil) of primary emulsion in the double emulsion of W1/O/W2 (W2 of skim milk) with 50–90 nm in size in 1–2 min of sonication time using 20 kHz US at 400 W and at an amplitude of 70% with 9% of polyglycerol polyricinoleate (PGPR) emulsifier. The encapsulation efficiency of B12 was 79.7–88.5%. The study has indicated that ultrasonic time and volume of W1/O emulsions (lower) are two important elements to consider when attempting to generate a double emulsion with a higher encapsulation efficiency of bioactives [136]. A similar study by Maghamian et al., 2021 [137] has demonstrated about 92% encapsulation efficiency of 0.2% glycyrrhizic acid (weakly polar bioactive) in skim milk double emulsion of 15 & 24% W1/O using 6–10% PGPR and 3 & 4 min of sonication time. The glycyrrhizic acid has various nutraceutical benefits including anti-inflammatory [138] and antiviral properties [139]. In a different study on emulsification of turmeric oil in skim milk, authors have demonstrated that stirring process sequentially followed by ultrasound has created stable emulsions within minimum emulsion droplet size than stirring process along with ultrasound treatment [140]. A study by Shanmugam & Ashokumar, 2014b [141], has shown the safety aspects of low frequency ultrasound in relation oxidative stability of flaxseed oil upon sonication. The conjugated diene (CD) values of skim milk based ready to drink emulsion with encapsulant as flaxseed oil remained constant until 8 days of storage at 4 ± 2 °C at all processing times between 1 and 8 min and until 176 W of treatment and was summation of CD values of individual raw material, the skim milk and flaxseed oil. The usage of low frequency ultrasound at optimum nominal applied power and presence of US-induced partially denatured proteins has shown the protective effect against oxidation [141]. A similar study, showed no changes to peroxide value and fatty acid profile after sono-encapsulation of mono unsaturated fatty acids rich olive oil (7–15%) in skim milk using 24 kHz at intensity of 85 W/cm^2^ [139]. In a different study by Silva et al., 2020a [142] 10% grape seed oil was encapsulated in milk protein solutions of 3% with altering concentrations of casein and whey protein such as 60:40, 50:50 and 40:60 using low frequency ultrasound and the digestibility of emulsion followed by free fatty acid (FFA) release were reported. It is indicated that the composition and structure of milk proteins at the oil-water interface are crucial for gastric digestion, and the presence of native whey proteins at the interface decreased the rate of digestion in stomach while additional caseins enhance the rate of digestion in the stomach. However, in terms of release of the main parameter FFA, it was understood that emulsions with higher whey proteins namely, 40:60 released FFA faster than 60:40 and 50:50 milk protein solutions of casein and whey, respectively. It was highlighted the higher digestion of 60:40 and 50:50 samples in stomach led to increased surface area of emulsion droplets because of their higher flocculation and coalescence leading to decreased access to intestinal enzyme lipase and further the release of FFA. This information is vital in design of wall material for encapsulants in the delivery of nutraceutical bioactive compounds. The same authors Silva et al., 2020b, have also studied the emulsion stability of grape seed oil using different milk protein solutions of 60:40, 50:50 and 40:60 of casein and whey proteins using low frequency ultrasound at 89.1 J/mL. They have shown that emulsions with majorly whey proteins at the droplet surface increased stability by steric stabilization but emulsions with majorly casein proteins at the droplet surface showed instability due to depletion flocculation mechanism. But the same study has indicated that casein protein stabilized emulsions have more oxidative stability than the whey protein emulsions [143]. The same authors Silva et al., 2020c, have also found that if more whey proteins are present in aqueous phase of O/W emulsions than caseins, the native whey proteins and aggregates of whey are present on droplet surfaces of emulsions contributing to finer droplet sizes [144]. In another study, Silva et al., 2020d have found that the droplet size of primary emulsion (W1/O) did not affect the encapsulation efficiency of grape seed oil as well as droplet size of double emulsion W1/O/W2. However, it was controlled by the composition of W2 phase, i.e., the whey proteins [145].

Applications of HPP for encapsulation of bioactives in dairy beverages are seen not literature. However, there are few study models using milk proteins such as whey protein concentrate, whey protein isolate and sodium caseinate or caseins as emulsifiers in dispersing oil in any polar medium by employing HPP. Those studies have indicated the changes to the emulsifying capacity of such dairy protein and their functionality using HPP [146]. β-Lactoglobulin undergoes structural unfolding and conformational changes after HPP treatment, which increases sulfhydryl-disulfide interactions and exposes free sulfhydryl groups to the aqueous medium. Thus, that would hasten the polymerization of β-lactoglobulin, which is adsorbed at the interface between the oil and water phases of the emulsion. Therefore, the polymerization of proteins adsorbed at the interface area to create a strong viscoelastic film layer helps stabilise emulsion systems [147]. Similarly, in the study by Kresic et al., 2006 [148], the improvement in protein functionality is reported for WPI at pressures of 600 Mpa with improvement in solubility and surface hydrophobicity. Also, they have highlighted that prolonged exposure period resulted in decrease in functional properties of the proteins. Other authors have also reported decrease in emulsion activity of WPC at higher pressure and longer processing time such as 800 MPa and 20 min [149]. Baier et al., 2015 [150] has also reported decrease in emulsion activity of micellar casein to slight lesser extent in comparison to WPI even at process in in low temperature conditions like room temperature and for 20 min of processing time. In a different study, a model emulsion with oil of 20% employing WPI as emulsifier has reported unfolding of proteins at processing conditions of 500 MPa resulting in increase in viscosity of samples with good emulsion stability [151]. Several model emulsions which may not have the properties of beverage enriched with nutraceuticals because of the higher levels of emulsified component from 15% to 45% such as sunflower oil, soyabean, peanut oil, corn oil, fish oil n-tetradecane have been reported in a review using HPP of 200–800 MPa used milk proteins along with usage of various stabilizers like pectin, xanthan, chitosan, locust bean gum, guar gum, etc. and emulsifiers like polyoxyethylene sorbitan monolaurate, soy lecithin at various processing times. More positive results were shown in terms of emulsion stability than negative effects of processing [152]. Therefore, a huge opportunity of encapsulation of bioactives in dairy beverages remains unexplored, posing a large opportunity for research in the area of HPP as emulsification tool.

## 6. Conclusions & Future Directions

Encapsulation of nutraceuticals using US and HPP technologies on producing yoghurt and beverage products can be considered as one of the emerging areas in dairy science. Sensitivity of nutraceuticals is highly dependent on the various intrinsic and extrinsic factors such as the properties of food products that the nutraceutical is going to encapsulate (food structure, food composition, physicochemical properties of food) and several environmental factors (processing method, processing conditions and storage conditions). Both US and HPP technologies are considered as non-destructive, energy efficient and emerging technologies in dairy industry. It provides several benefits in improving process and product characteristics while preserving the nutrients and organoleptic qualities of dairy products and this review is aiming on yoghurt and beverage products.

Optimization of process parameters during the application of US or HPP methods is crucial as all the parameters may need to be optimized based on the composition of the milk base, processes involved, and type of the yoghurt/beverage being produced. Thus, this review article provides knowledge on how the US or HPP process parameters can optimize as necessarily for different product characteristics. However, more research and modelling are in need to establish the relationship between the process parameters and product qualities.

Both US and HPP technologies have potential to significantly reduce the processing costs due to its energy efficient nature, easy to scale up production and easy operation. However, scaling up has not been picked up quickly as compared to lab scale research due to its high initial capital establishment costs. Therefore, this lack of up take by the industry needs to be addressed to provide more industrial scale ups by providing more cost friendly industrial equipment. In addition, more research is needed to integrate ultrasonication or high-pressure processing into the current production lines. Most of the research are based on lab scale and batch set ups. Thus, how these technologies are effectively work in improving the qualities of yoghurt and beverages in flow through systems are needed to replicate the production set up in a factory.

The over processing disadvantages such as off flavours and colour defects associated with both ultrasound and high-pressure processing need to be carefully monitored in terms of the milk base used, parameters used and what other measures can be in cooperated to minimise those effects. Even though, there are many yoghurt or dairy emulsions have developed with the addition of nutraceuticals, probiotics and prebiotics, how these components interact with other milk components affected by ultrasound or high-pressure processing is not yet sufficiently studied. The correlations of how these bioactives/nutraceuticals increase their bioavailability and bioactivity under the influence of these technologies is needed.

## Figures and Tables

**Table 1:** The effect of ultrasound on the rheological properties of yoghurt gels.

Ultrasound Conditions	Effect of Ultrasound on the Rheological Properties	References
20 kHz frequency, 90 W, 225 W and 450 W for 1, 6, 8 and 10 min	Sonication before inoculation resulted improvements in viscosity and water holding capasity with reduced syneresis at higher power levels of 180–450 W for 6 and 8 min. Sonication after inoculation had no advantageous effect on syneresis.	Wu et al., 2000 [18]
20 kHz frequency, 90 W, 225 W and 315 W for 3 and 6 min	Resulted higher water holding capasity compared to the control sample. Highest amplitude and treatment time combination (315 W and 6 min) provided highest water holding capasity of 66.18%.	Șengül et al., 2009 [31]
20 kHz frequency, 150, 262, 375, 562 and 750 W for 10 min skim milk	Resulted higher viscosity compared to the control sample. Kinetically, sonication shorten the duration of the lag phase of viscosity and increased the maximum rate of viscosity increase as increases the amplitude.	Sfakianakis et al., 2015b [27]
20 kHz frequency, 40 °C temperature, 2 kg/cm^2^ pressure for 12 s	Resulted strong gel structure with improved textural properties. Sonicated yoghurts were harder, more adhesive, had higher gumminess and chewiness compared to the control sample. Water holding capacity was higher in sonicated sample showing 40% less serum separation than the control. Gʹ value also ~50% higher in sonicated samples.	Vercet et al., 2002b [32]
24 kHz frequency, 400 W output power at 72 °C	When the milk contains 1.5 or 3.5% fat, 2-fold increase in water holding capacity and 25% higher Gʹ were observed with increased viscosity and firmness. Sonicated gel had a honeycomb-like network with a more porous microstructure.	Riener et al., 2009c [52]
20 kHz frequency, 400 W for 10 min at 45 °C	Improvements in viscosity and water holding capasity were observed with reduced syneresis.	Riener et al., 2010 [52]
22.5 kHz frequency and 50 W output power, uncontrolled temperature for whole milk	5 min treatment resulted Higher storage module (G′) of (~500 Pa) than the controlled sample (10 Pa) and shorter gelation times (45 min) compared to the untreated sample (85 min). About 40% of whey proteins were denatured and homogenised the fat globules resulting 50% increase in fat globule surface area. Prolong sonication (>30 min) resulted reduction in (G′) to 175 Pa and 100% denaturation of whey with greater aggregation.	Nguyen and Anema, 2017 [53]
22.5 kHz frequency and 50 W output power, controlled temperature for whole milk	Increase in storage module (Gʹ) were observed up to 60 °C with subsequent reduction upon higher temperature and processing times. At 20 and 40 °C for 30 min, minor reduction (10 min) in gelation time was observed.	Nguyen and Anema, 2017 [53]
20 kHz frequency and 200 W power during skim milk fermentation when pH was 5.8 to 5.1.	Sonication reduced the gel firmness by 80% and torque to break the gel by 75%. Stirred yoghurt prepared from sonicated samples by agitating the gel had smoother texture with less aggregates and reduced viscosity due to the less cohesive structure and more compact microgel particles.	Körzendörfer et al., 2019 [54]
20 kHz frequency, 200 W power for 5 S for milk concentrates after the fermentation process	Stirred yoghurt produced from set gels at pH 4.8 and 5.0 resulted softer gels, less grainy appearance, reduced viscosity and water holding capacity compared to the set gels at pH 4.6.	Koerzendoerfer and Hinrichs, 2019 [55]
24 kHz frequency, 100, 125 and 150 W for 15 min and 70 °C (5 min) whole milk	Sonicated yoghurt drink samples showed higher viscosity and reduced serum seperation. No serum seperation was observed under the 150 W power. Sonication treatment had no effect on proximate composition or color of the yoghurt.	Gursoy et al., 2016 [56]
20 kHz frequency, 40% amplitude for WPI solutions	Resulted higher water holding capacity, gel strength and gel firmness with dense and uniform gel networks.	Shen et al., 2017 [57]
20 kHz frequency, 750 W power for 20 min for whey protein concentrates	Resulted better elastic gelling properties compared to the control sample.	Arzeni et al., 2012 [58]

## Data Availability

All data are included in the main text.
